# Androgen Deprivation Therapy and Outcomes After Radiation Therapy in Black Patients With Prostate Cancer

**DOI:** 10.1001/jamanetworkopen.2024.15911

**Published:** 2024-06-10

**Authors:** Kylie M. Morgan, Paul Riviere, Tyler J. Nelson, Kripa Guram, Leah N. Deshler, Daniel Sabater Minarim, Elizabeth A. Duran, Matthew P. Banegas, Brent S. Rose

**Affiliations:** 1 Department of Radiation Medicine and Applied Sciences, University of California, San Diego, La Jolla; 2 Veterans Health Affairs San Diego Health Care System, La Jolla, California; 3 Center for Health Equity, Education and Research, Department of Radiation Medicine and Applied Sciences, University of California San Diego Health, La Jolla; 4 Department of Urology, University of California San Diego Health, La Jolla

## Abstract

**Importance:**

Prostate cancer in Black men compared with White men may be more sensitive to radiation therapy resulting in better outcomes in equal-access settings. The outcomes of androgen-deprivation therapy (ADT) vs radiation therapy itself remains uncharacterized.

**Objectives:**

To quantify any outcome modification by receipt of ADT on the association between Black race and prostate cancer outcomes following radiation therapy.

**Design, Setting, and Participants:**

This was a retrospective, nationwide cohort study of Black and White patients treated in the US Veterans Healthcare system between 2000 and 2020 receiving definitive radiation for localized prostate cancer. Data were analyzed from January 2000 to December 2020.

**Exposure:**

Patient self-identified race and use of ADT defined as any gonadotrophin-releasing hormone agonist or antagonist prescription within 6 months of radiation.

**Main Outcomes and Measures:**

Biochemical recurrence (BCR) from time of completion of radiation therapy (prostate-specific antigen nadir plus 2 ng/mL) and development of metastatic disease or prostate cancer mortality (PCSM) from time of recurrence.

**Results:**

A total of 26 542 patients (8716 Black men with median [IQR] age of 64 [59-69] years and 17 826 White men with median [IQR] age of 67 [62-72] years) received definitive radiation therapy for nonmetastatic prostate cancer and had complete staging and follow-up data. A total of 5144 patients experienced BCR (3384 White and 1760 Black patients). The cumulative incidence of BCR at 10 years was not significantly different between Black and White men (1602 [22.14%] vs 3099 [20.13%], respectively) with multivariable hazard ratio (HR) of 1.03 (95% CI, 0.97-1.09; *P* = .33). In men receiving ADT, Black men had an HR for BCR of 0.90 (95% CI, 0.82-0.99; *P* = .03) compared with White men, and in men not receiving ADT, Black men had an HR of 1.13 (95% CI, 1.05-1.22; *P* = .002). Black race was associated with a decreased risk of developing metastatic disease (HR, 0.90; 95% CI, 0.82-0.98; *P* = .02) or PCSM (subdistribution HR, 0.72; 95% CI, 0.63-0.82; *P* < .001) from time of biochemical recurrence.

**Conclusions and Relevance:**

Black patients treated with radiation appear to specifically benefit from the addition of ADT with regard to biochemical control. Additionally, BCR in Black men results in a lower rate of metastatic disease and death from prostate cancer. Future analyses of radiosensitivity in Black men should evaluate for the possibility of outcome modification by ADT.

## Key Points

**Question:**

Do Black patients undergoing radiation therapy for prostate cancer demonstrate differential sensitivity to androgen deprivation therapy (ADT) compared with White patients?

**Findings:**

This nationwide, multicenter cohort study of 26 542 patients found that Black patients receiving ADT for prostate cancer (as compared with White patients) had a significantly lower risk of biochemical recurrence, but when not receiving ADT, had a greater risk of biochemical recurrence than White patients, a significant treatment interaction.

**Meaning:**

These findings suggest that differential response to ADT by race may contribute to the better outcomes among Black men undergoing radiation therapy for prostate cancer.

## Teaser

This cohort study evaluates whether receipt of androgen deprivation therapy is associated with outcomes among Black men with prostate cancer who underwent radiation therapy.

## Introduction

Recent data have led researchers to hypothesize that prostate cancer arising in Black patients may be more radiosensitive than in non-Hispanic White patients. A meta-analysis of Black and non-Hispanic White patients with prostate cancer treated within 7 randomized clinical trials with definitive radiotherapy and a variety of androgen-deprivation therapy (ADT) regimens found that Black patients had a lower subdistribution hazard ratio (sHR) of biochemical failure (BCF), distant metastases (DM), and prostate cancer–specific mortality (PCSM).^1^ One possible explanation is that non-Hispanic Black patients may have increased sensitivity to ADT, which would result in better outcomes both when ADT is used as a part of initial definitive radiotherapy and as systemic therapy after biochemical recurrence. Non-Hispanic Black men with prostate cancer have greater prostate cancer androgen receptor expression,^2^ more variations in the AR receptor,^3^ and appear to have better survival outcomes than non-Hispanic White men when treated with abiraterone in the metastatic castration-resistant setting.^4^

This study sought to better understand the mechanisms leading to improved prostate cancer outcomes in Black men receiving equal access to curative-intent radiation therapy for prostate cancer. We assembled a cohort of Black and White patients with localized prostate cancer treated within the US Veterans Healthcare system (VA) with definitive radiation therapy stratified by concurrent ADT prescription and further studying DM and survival in the patients developing biochemical recurrence (BCR) following treatment.

## Methods

### Data Source

This retrospective cohort study used data in the VA national electronic health record, the Corporate Data Warehouse, and the VA Central Cancer Registry accessed through the VA Informatics and Computing Infrastructure (VINCI). Data including patient demographics, clinical notes, laboratory results, pathology reports, and cancer treatment and outcomes were analyzed.

The VA Central Cancer Registry conforms to standards set by the North American Association of Central Cancer Registries for detecting and reporting incident cancer cases and treatments. The San Diego VA institutional review board approved this study. Informed consent was waived due to the nature of the study and the minimal risk posed to patients. This study followed the Strengthening the Reporting of Observational Studies in Epidemiology (STROBE) reporting guideline.^5^

### Study Population

We identified 44 579 patients with nonmetastatic prostate cancer and a prostate-specific antigen (PSA) test result within 180 days before diagnosis who received definitive external beam radiation therapy between 2000 and 2020. A total of 15 495 patients with unknown information in covariates were excluded. Patients were identified as Black or White based on self-identified race; patients without a self-identified race of Black or White documented in the electronic health record were excluded (2542 patients). All patients were followed up until death or last follow-up with a VA clinician.

We obtained information on ethnicity, age, year of diagnosis, marital status, employment status, tobacco history, and alcohol history. The Charlson comorbidity index score was calculated from comorbid conditions present in the year leading up to the diagnosis of prostate cancer.

Cancer characteristics, including Gleason score, PSA, and clinical tumor and lymph node TNM staging at diagnosis, were captured. PSA values were obtained from laboratory results rather than registry records. Cancer risk groups were as defined by the National Comprehensive Cancer Network (NCCN) risk stratification.^6^

ADT exposure was defined as any prescription of a gonadotrophin-releasing hormone receptor agonist or antagonist within the 6 months before or after the initiation of radiation therapy. Duration of ADT was not captured. History of pelvic or prostate magnetic resonance imgaing (MRI) was obtained from the VINCI Prostate Cancer Data Core. Brachytherapy was defined as nonexternal beam radiation therapy.

### Outcomes

The end points of the study included treatment start to BCR, defined as PSA nadir plus 2 ng/mL (to convert to μg/L, multiply by 1.0) after completion of definitive radiation therapy^7^ and treatment start to development of metastases, as identified in the PCa Data Core using natural language processing and previously validated methods.^8^ In the cohort of patients who did experience BCR, patients were analyzed for time from biochemical recurrence to development of metastases and overall survival from time of biochemical recurrence. Cause of death was ascertained using data from the National Death Index (NDI). For each analysis, patients who did not experience an event of interest were censored either at date of last follow up or on December 31, 2020 (last date of available NDI data), whichever occurred first.

### Statistical Analysis

To compare categorical variables between Black and White men, we used the χ^2^ test. For continuous outcomes, we used the Wilcoxon rank-sum test. We used cumulative incidence function curves to visualize the rates of development of metastases from time of BCR as well as rate of BCR or development of metastases from time of completion of radiation therapy (eFigure 1 in Supplement 1).

To evaluate the association of various factors with the outcomes of treatment start to biochemical recurrence, treatment start to development of metastases, biochemical recurrence to development of metastases, and biochemical recurrence to death, we used Cox proportional hazards models with death from noncancer causes as a competing event. These models allowed us to estimate hazard ratios (HRs) and associated 95% CIs, adjusting for all variables listed in Table 1. Fine-Gray regression technique was used for competing risk analysis in PCSM, accounting for noncancer death as a competing event; these results were reported with SHR and 95% CIs. All statistical tests were 2-sided, with significance defined as *P* < .05, and analysis was performed using R Studio version 3.5.1 (R Project for Statistical Computing). Data were analyzed from January 2000 to December 2020.

**Table 1.** Demographics

Variable	Participants, No. (%)		*P* value
	Black (n = 8716)	White (n = 17 826)	
Age, median (IQR), y	64 (59-69)	67 (62-72)	<.001
Year of diagnosis			
2000-2005	1892 (21.7)	4282 (24.0)	<.001
2006-2010	498 (5.7)	1117 (6.3)	
2011-2015	2811 (32.3)	6057 (34.0)	
2016-2020	3515 (40.3)	6370 (35.7)	
Ethnicity			
Hispanic or Latino	154 (1.8)	1074 (6.0)	<.001
Not Hispanic or Latino	8469 (97.2)	16 532 (92.7)	
Unknown	93 (1.1)	220 (1.2)	
Gleason score			
Gleason ≤6	2707 (31.1)	5774 (32.4)	<.001
Gleason 7	4230 (48.5)	8102 (45.5)	
Gleason ≥8	1779 (20.4)	3950 (22.2)	
PSA, median (IQR), ng/mL	7.2 (5.3-11.9)	6.9 (5.1-10.4)	<.001
Clinical N staging			
N0	8683 (99.6)	17751 (99.6)	.69
N1	33 (0.4)	75 (0.4)	
Clinical T stage			
T1	6496 (74.5)	11 792 (66.2)	<.001
T2	2005 (23.0)	5496 (30.8)	
T3	195 (2.2)	516 (2.9)	
T4	20 (0.2)	22 (0.1)	
Charlson comorbidity			
0	3905 (44.8)	8282 (46.5)	<.001
1	3832 (44.0)	8332 (46.7)	
≥2	979 (11.2)	1212 (6.8)	
Marital status			
Married	3521 (40.4)	9795 (54.9)	<.001
Not married	5195 (59.6)	8031 (45.1)	
Employment status			
Currently employed	1259 (14.4)	2609 (14.6)	.69
Not employed	7457 (85.6)	15 217 (85.4)	
Smoking status			
Nonsmoker	6206 (71.2)	13 882 (77.9)	<.001
Current	2510 (28.8)	3944 (22.1)	
Alcohol history			
None	5934 (68.1)	12 081 (67.8)	.62
Current	2782 (31.9)	5745 (32.2)	
MRI			
None	8238 (94.5)	17 182 (96.4)	<.001
Yes	478 (5.5)	644 (3.6)	
ADT treatment			
None	5053 (58.0)	10 680 (59.9)	.003
Yes	3663 (42.0)	7146 (40.1)	
Brachytherapy			
None	7791 (89.4)	14 563 (81.7)	<.001
Yes	925 (10.6)	3263 (18.3)	

Abbreviations: ADT, androgen deprivation therapy; MRI, magnetic resonance imaging; N, lymph node; PSA, prostate specific antigen; T, tumor.

## Results

### Baseline Characteristics

The final cohort included 26 542 men, of whom 8716 were Black and 17 826 were White (Table 1). Compared with White men, Black men were more likely to report current smoking (2510 patients [28.8%] vs 3994 [22.1%]), less likely to be married (3521 patients [40.4%] vs 9795 patients [54.9%]), and had a lower median (IQR) age at diagnosis (64 [59-69] years vs 67 [62-72] years). Additionally, the prevalence of medical comorbidities (Charlson comorbidity index ≥2) was higher in Black men than White men (979 patients [11.2%] vs 1212 patients [6.8%]). Black men had a higher median (IQR) PSA (7.24 [5.27-11.89] ng/mL vs 6.90 [5.10-10.40] ng/mL) at diagnosis compared with White men. Black patients were equally as likely as White patients to present with NCCN high-risk disease. Black men were more likely to receive ADT as a component of therapy (3663 patients [42.0%] vs 7146 patients [40.1%]), more likely to have a history of prostate or pelvic MRI (478 patients [5.5%] vs 644 patients [3.6%]), and less likely to receive brachytherapy (925 patients [10.6%] vs 3263 patients [18.3%]) than White men.

### Biochemical Recurrence

A total of 5144 patients experienced BCR (3384 White men and 1760 Black men). The cumulative incidence of BCR at 10 years was not significantly different between Black men and White men (1602 patients [22.14%] vs 3099 patients [20.13%], respectively) (eFigure 1 in Supplement 1). In a multivariable Cox proportional hazards model, Black race was not associated with an increased risk of BCR (HR, 1.03; 95% CI, 0.97-1.09; *P* = .33) (Table 2). We found a statistically significant interaction between race and ADT on BCR (*P* = .006). Stratifying by whether patients had received ADT as a part of their radiation therapy course, in men receiving ADT, Black men had an HR of 0.90 (95% CI, 0.82-0.99; *P* = .03) as compared with White men, and in men not receiving ADT, Black men had an HR of 1.13 (95% CI, 1.05-1.22; *P* = .002) (Table 3).

**Table 2.** Time to Recurrence or Metastases

Variable	Biochemical Recurrence		Metastases	
	HR (95% CI)	*P* value	HR (95% CI)	*P* value
Race				
Black	1.03 (0.97-1.09)	.33	0.90 (0.82-0.98)	.02
White	1 [Reference]	NA	1 [Reference]	NA
Ethnicity				
Hispanic or Latino	0.99 (0.87-1.12)	.85	0.87 (0.71-1.07)	.19
Not Hispanic or Latino	1 [Reference]	NA	1 [Reference]	NA
Unknown	1.25 (0.97-1.61)	.08	1.72 (1.22-2.41)	.002
Charlson Comorbidity Index				
0	1 [Reference]	NA	1 [Reference]	NA
1	0.90 (0.85-0.96)	<.001	0.67 (0.62-0.74)	<.001
≥2	0.95 (0.86-1.05)	.33	0.78 (0.66-0.91)	.002
Marital status				
Married	1 [Reference]	NA	1 [Reference]	NA
Not married	1.04 (0.99-1.10)	.15	1.10 (1.01-1.20)	.02
Employment status				
Employed	1 [Reference]	NA	1 [Reference]	NA
Not employed	0.94 (0.87-1.02)	.15	0.92 (0.81-1.04)	.16
Alcohol history				
Current	1.02 (0.96-1.09)	.54	1.04 (0.95-1.14)	.36
None	1 [Reference]	NA	1 [Reference]	NA
Smoking				
Any	1.03 (0.96-1.10)	.40	1.02 (0.92-1.13)	.76
Nonsmoking	1 [Reference]	NA	1 [Reference]	NA
ADT treatment	0.75 (0.70-0.79)	<.001	0.96 (0.87-1.06)	.41
Gleason score				
Gleason 6	1 [Reference]	NA	1 [Reference]	NA
Gleason 7	1.35 (1.26-1.44)	<.001	1.50 (1.34-1.68)	<.001
≥Gleason 8	1.84 (1.69-2.00)	<.001	2.78 (2.45-3.16)	<.001
Age at diagnosis	0.99 (0.99-1.00)	.003	0.99 (0.99-1.00)	.04
PSA, ng/mL				
<10	1 [Reference]	NA	1 [Reference]	NA
10-20	1.53 (1.42-1.64)	<.001	1.50 (1.35-1.66)	<.001
>20	2.22 (2.05-2.41)	<.001	2.37 (2.11-2.66)	<.001
Clinical T				
T1	1 [Reference]	NA	1 [Reference]	NA
T2	1.23 (1.15-1.30)	<.001	1.41 (1.29-1.54)	<.001
T3	1.48 (1.30-1.70)	<.001	1.93 (1.62-2.29)	<.001
T4	4.55 (3.00-6.91)	<.001	7.54 (4.80-11.84)	<.001
Clinical N				
N0	1 [Reference]	NA	1 [Reference]	NA
N1	3.15 (2.47-4.03)	<.001	4.85 (3.72-6.32)	<.001
Year of diagnosis				
2000-2005	1 [Reference]	NA	1 [Reference]	NA
2006-2010	0.82 (0.73-0.92)	<.001	0.98 (0.83-1.17)	.86
2011-2015	0.80 (0.75-0.86)	<.001	0.99 (0.89-1.11)	.88
2016-2020	0.71 (0.65-0.77)	<.001	1.16 (1.02-1.31)	.02
MRI				
No	1 [Reference]	NA	1 [Reference]	NA
Yes	0.99 (0.83-1.17)	.88	1.17 (0.94-1.46)	.16

Abbreviations: ADT, androgen deprivation therapy; HR, hazard ratio; MRI, magnetic resonance imaging; N, lymph node; NA, not applicable; PSA, prostate specific antigen; T, tumor.

**Table 3.** Biochemical Recurrence Stratified by Use of Androgen Deprivation Therapy (ADT) in Initial Therapy

Variable	ADT		No ADT	
	HR (95% CI)	P value	HR (95% CI)	P value
Race				
Black	0.90 (0.82-0.99)	.03	1.13 (1.05-1.22)	.002
White	1 [Reference]	NA	1 [Reference]	NA
Ethnicity				
Hispanic or Latino	0.94 (0.77-1.14)	.51	1.05 (0.89-1.25)	.56
Not Hispanic or Latino	1 [Reference]	NA	1 [Reference]	NA
Unknown	1.64 (1.15-2.33)	.01	1.01 (0.71-1.45)	.95
Charlson				
0	1 [Reference]	NA	1 [Reference]	NA
1	0.84 (0.77-0.92)	<.001	0.95 (0.88-1.02)	.16
≥2	0.96 (0.82-1.12)	.62	0.96 (0.83-1.09)	.52
Marital status				
Married	1 [Reference]	NA	1 [Reference]	NA
Not married	1.04 (0.95-1.14)	.36	1.03 (0.96-1.11)	.37
Employment status				
Employed	1 [Reference]	NA	1 [Reference]	NA
Not employed	0.97 (0.85-1.10)	.63	0.93 (0.84-1.03)	.18
Alcohol history				
Current	1.05 (0.96-1.15)	.25	1.00 (0.92-1.08)	.93
None	1 [Reference]	NA	1 [Reference]	NA
Smoking				
Any	1.01 (0.91-1.12)	.92	1.04 (0.95-1.14)	.41
Nonsmoking	1 [Reference]	NA	1 [Reference]	NA
Gleason score				
Gleason 6	1 [Reference]	NA	1 [Reference]	NA
Gleason 7	1.17 (1.02-1.33)	.02	1.46 (1.35-1.58)	<.001
≥Gleason 8	1.60 (1.40-1.83)	<.001	1.98 (1.75-2.25)	<.001
Age at diagnosis	0.99 (0.98-0.99)	<.001	1.00 (0.99-1.00)	.78
PSA, ng/mL				
<10	1 [Reference]	NA	1 [Reference]	NA
10-20	1.50 (1.35-1.66)	<.001	1.61 (1.47-1.76)	<.001
>20	2.44 (2.20-2.71)	<.001	1.90 (1.63-2.21)	<.001
Clinical T				
T1	1 [Reference]	NA	1 [Reference]	NA
T2	1.22 (1.12-1.34)	<.001	1.23 (1.14-1.33)	<.001
T3	1.61 (1.37-1.88)	<.001	1.28 (0.97-1.70)	.08
T4	4.50 (2.79-7.24)	<.001	4.36 (1.79-10.63)	.001
Clinical N				
N0	1 [Reference]	NA	1 [Reference]	NA
N1	3.90 (2.98-5.09)	<.001	1.41 (0.74-2.67)	.30
Year of diagnosis				
2000-2005	1 [Reference]	NA	1 [Reference]	NA
2006-2010	0.89 (0.74-1.06)	.19	0.78 (0.67-0.91)	.001
2011-2015	0.91 (0.82-1.02)	.11	0.75 (0.68-0.81)	<.001
2016-2020	1.02 (0.90-1.16)	.77	0.55 (0.49-0.61)	<.001
MRI				
No	1 [Reference]	NA	1 [Reference]	NA
Yes	1.01 (0.82-1.25)	.92	0.80 (0.59-1.08)	.14

Abbreviations: HR, hazard ratio; MRI, magnetic resonance imaging; N, lymph node; NA, not applicable; PSA, prostate specific antigen; T, tumor.

### Metastases

Of the total cohort, 2224 patients developed metastases (1511 White men and 713 Black men). The cumulative incidence of metastases at 10 years was not significantly different between Black men and White men (8.13% vs 8.46%) (eFigure 1 in Supplement 1). In the multivariable Cox proportional hazards model, Black race was associated with a decreased risk of developing metastatic disease (HR, 0.90; 95% CI, 0.82-0.98; *P* = .03) (Table 2). There was no significant interaction between race and ADT for time-to-metastatic disease.

### BCR to Metastases

Among the patients who experienced BCR, 2224 patients went on to develop metastatic disease. Among these, 770 patients had metastatic disease at time of diagnosis of BCR. The cumulative incidence of metastases at 10 years after BCR was significantly lower in Black men than in White men (46.02% vs 51.05%) (Figure); this resulted in a multivariable HR of 0.86 (95% CI, 0.78-0.94; *P* < .001) (eTable in Supplement 1). There was no significant interaction between race and ADT with respect to development of metastatic disease from time of biochemical recurrence.

**Figure.**
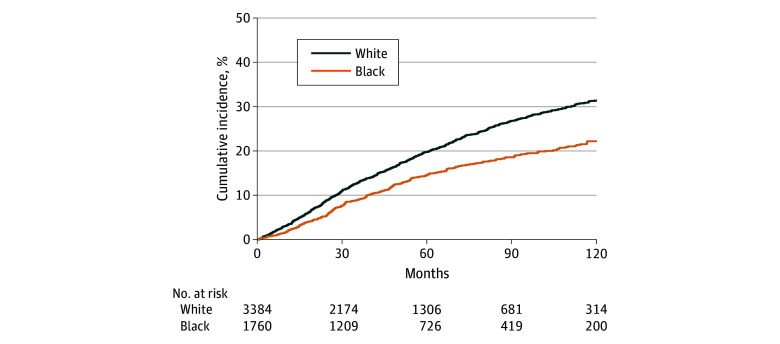
Cumulative Incidence of Metastases After Biochemical Recurrence

### BCR to Death From Prostate Cancer

Among the subset of patients experiencing BCR, 2897 patients died (2017 White men and 880 Black men). Among these, 1144 patients died due to cancer (835 White men and 309 Black men). In the multivariable Cox proportional hazards model, Black race was associated with a decreased risk of death (HR, 0.81; 95% CI, 0.75-0.88; *P* < .001) (eFigure 2 in Supplement 1). When studying PCSM in competing-risk analysis, Black race was associated with a lower risk of PCSM (SHR, 0.72; 95% CI, 0.63-0.82; *P* < .001) (Table 4). There was no significant interaction between race and ADT and time to PCSM.

**Table 4.** Prostate Cancer–Specific Mortality From Recurrence

Variable	SHR (95% CI)^a^	*P* value
Race		
Black	0.73 (0.64-0.82)	<.001
White	1 [Reference]	NA
Ethnicity		
Hispanic or Latino	0.94 (0.75-1.20)	.63
Not Hispanic or Latino	1 [Reference]	NA
Unknown	1.69 (1.11-2.56)	.01
Charlson		
0	1 [Reference]	NA
1	0.73 (0.65-0.82)	<.001
≥2	0.69 (0.56-0.86)	<.001
Marital status		
Married	1 [Reference]	NA
Not married	0.98 (0.88-1.10)	.78
Employment status		
Employed	1 [Reference]	NA
Not employed	0.96 (0.81-1.13)	.60
Alcohol history		
Current	0.95 (0.85-1.08)	.42
None	1 [Reference]	NA
Smoking		
Any	1.02 (0.90-1.18)	.71
Nonsmoking	1 [Reference]	NA
ADT	1.28 (1.13-1.46)	<.001
Gleason score		
Gleason 6	1 [Reference]	NA
Gleason 7	1.33 (1.14-1.56)	<.001
≥Gleason 8	2.13 (1.79-2.54)	<.001
Age at diagnosis	1.00 (1.00-1.01)	.23
PSA, ng/mL		
<10	1 [Reference]	NA
10-20	1.10 (0.96-1.26)	.19
>20	1.12 (0.95-1.31)	.17
Clinical T		
T1	1 [Reference]	NA
T2	1.24 (1.10-1.40)	<.001
T3	1.68 (1.37-2.07)	<.001
T4	1.74 (0.90-3.38)	.10
Clinical N		
N0	1 [Reference]	NA
N1	0.71 (0.44-1.14)	.16
Year of diagnosis		
2000-2005	1 [Reference]	NA
2006-2010	1.22 (1.00-1.48)	.05
2011-2015	0.98 (0.86-1.11)	.72
2016-2020	0.97 (0.82-1.15)	.72
MRI		
No	1 [Reference]	NA
Yes	0.72 (0.49-1.08)	.11

Abbreviations: ADT, androgen deprivation therapy; N, lymph node; NA, not applicable; PSA, prostate specific antigen; SHR, subdistribution hazard ratio; T, tumor.

^a^ Fine-Gray regression of prostate cancer specific mortality accounting for noncancer death as a competing event.

## Discussion

In this study, we found that Black patients with localized prostate cancer treated with definitive radiotherapy in an equal-access health care setting have overall equivalent rates of biochemical progression compared with White men. However, when stratifying based on ADT as a part of initial therapy, Black men receiving ADT had better biochemical control (HR, 0.90; 95% CI, 0.82-0.99) than their White counterparts, and when not receiving ADT, they had worse biochemical control than White men (HR, 1.13; 95% CI, 1.05-1.22). Among patients who experienced biochemical recurrence, Black patients had a lower rate of development of metastatic disease (HR, 0.86; 95% CI, 0.78-0.94) or dying from prostate cancer (SHR, 0.72; 95% CI, 0.63-0.82) and better overall survival than White men (HR, 0.81; 95% CI, 0.75-0.88). Together, these findings suggest that ADT responsiveness may play an important role in the improved outcomes in Black men undergoing definitive radiotherapy.

These findings come at a time of evolving understanding of racial disparities in prostate cancer outcomes for Black patients. It is likely that there are biologic mechanisms to explain the increased incidence and earlier onset of prostate cancer in Black men compared with White men.^3,9,10,11^ In the general US population, Black men have worse overall and prostate cancer–specific survival from time of diagnosis.^12^ However, recent studies suggest that this latter finding of inferior cancer-specific survival is in large part driven by access to care and therapeutic utilization.^13,14,15^

Separately, investigations of the genomics of prostate cancer have led to putative mechanistic explanations for a possible higher radiosensitivity of prostate cancer in non-Hispanic Black men particularly. In samples from prostatectomy pathology, non-Hispanic Black men have a higher radiation sensitivity index^16^ than non-Hispanic White patients, and also better postoperative radiotherapy outcome scores.^9,17^ Clinically, a patient-level meta-analysis found that Black men in clinical trials may have lower rates of biochemical recurrence, metastasis, and PCSM compared with matched White patients.^1^ Specifically, the most important outcome was a reduction in developing metastasis or dying of prostate cancer (rather than for the biochemical recurrence end point), suggesting that race was associated with outcomes beyond just initial response to definitive radiotherapy. While there are other potential contributing factors to these findings, such as younger age at diagnosis in Black men, the consistently observed large effect size in that meta-analysis and the current study suggests that differential sensitivity to prostate cancer–directed therapy may help to explain these outcomes in Black patients.

In our study, we found that that there is a significant interaction between Black race and receipt of ADT on biochemical progression and recurrence in Black men treated with radiation, which could partially explain the result of the meta-analysis,^1^ as that study did not perform stratification based on ADT. Our finding of improved MFS and PCSM after BCR in Black men raises questions about whether the improved prostate cancer outcomes in Black patients in equal-access settings was a radiation-specific outcome. Alternatively, a difference in the natural history of relapsed prostate cancer or differential sensitivity to postrelapse therapies (ie, ADT) could be contributors.

### Limitations

Our study has several limitations. First, due to the use of nonrandomized, retrospective data it is possible for biases between Black and White selection of definitive radiotherapy (as opposed to prostatectomy) to result in unmeasured confounding. Additionally, the size of this cohort allowed for detection of statistically significant outcomes that may not be clinically meaningful. Separately, our cohort does not have associated genomic data, and thus we were unable to explore if specific genomic variants (including radiosensitivity markers) are driving the differential outcomes in the postbiochemical recurrent setting. Finally, because prescription of ADT is associated with prostate cancer risk stratification (as well as prognostication of competing mortality), this study could not determine if there was a risk-group specific outcome of these findings. The study of ADT response by race in specific risk groups remains a subject for future investigation.

## Conclusions

In this cohort study of patients experiencing BCR following definitive radiation therapy, we found that there may be a race-specific outcome of ADT that could be contributing to the differential outcomes in Black men receiving definitive radiation therapy for prostate cancer. Further study of this is warranted to validate these findings, and if confirmed, to further characterize the biologic mechanisms responsible for this.

## Notes

Supplement 1. **eTable. **Cox Regression of Development of Metastases From Time of Biochemical Recurrence **eFigure 1. **Prostate Cancer Outcomes in all Patients **eFigure 2. **Kaplan Meier

Supplement 2.
Data Sharing Statement

## References

[1] Ma TM, Romero T, Nickols NG, . Comparison of response to definitive radiotherapy for localized prostate cancer in Black and White men: a meta-analysis. JAMA Netw Open. 2021;4(12):e2139769. doi:10.1001/jamanetworkopen.2021.39769 34964855 PMC8717118

[2] Gaston KE, Kim D, Singh S, Ford OH III, Mohler JL. Racial differences in androgen receptor protein expression in men with clinically localized prostate cancer. J Urol. 2003;170(3):990-993. doi:10.1097/01.ju.0000079761.56154.e5 12913756

[3] Mahal BA, Alshalalfa M, Kensler KH, . Racial differences in genomic profiling of prostate cancer. N Engl J Med. 2020;383(11):1083-1085. doi:10.1056/NEJMc2000069 32905685 PMC8971922

[4] Marar M, Long Q, Mamtani R, Narayan V, Vapiwala N, Parikh RB. Outcomes among African American and non-Hispanic White men with metastatic castration-resistant prostate cancer with first-line abiraterone. JAMA Netw Open. 2022;5(1):e2142093. doi:10.1001/jamanetworkopen.2021.42093 34985518 PMC8733836

[5] Ghaferi AA, Schwartz TA, Pawlik TM. STROBE Reporting Guidelines for Observational Studies. JAMA Surg. 2021;156(6):577-578. doi:10.1001/jamasurg.2021.0528 33825815

[6] Schaeffer EM, Srinivas S, Adra N, . NCCN guidelines(r) insights: prostate cancer, version 1.2023. J Natl Compr Canc Netw. 2022;20(12):1288-1298. doi:10.6004/jnccn.2022.0063 36509074

[7] Roach M III, Hanks G, Thames H Jr, . Defining biochemical failure following radiotherapy with or without hormonal therapy in men with clinically localized prostate cancer: recommendations of the RTOG-ASTRO Phoenix Consensus Conference. Int J Radiat Oncol Biol Phys. 2006;65(4):965-974. doi:10.1016/j.ijrobp.2006.04.029 16798415

[8] Alba PR, Gao A, Lee KM, . Ascertainment of veterans with metastatic prostate cancer in electronic health records: demonstrating the case for natural language processing. JCO Clin Cancer Inform. 2021;5:1005-1014. doi:10.1200/CCI.21.00030 34570630

[9] Awasthi S, Berglund A, Abraham-Miranda J, . Comparative genomics reveals distinct immune-oncologic pathways in African American men with prostate cancer. Clin Cancer Res. 2021;27(1):320-329. doi:10.1158/1078-0432.CCR-20-2925 33037017 PMC8042600

[10] Saunders EJ, Kote-Jarai Z, Eeles RA. Identification of germline genetic variants that increase prostate cancer risk and influence development of aggressive disease. Cancers (Basel). 2021;13(4):760. doi:10.3390/cancers13040760 33673083 PMC7917798

[11] Amundadottir LT, Sulem P, Gudmundsson J, . A common variant associated with prostate cancer in European and African populations. Nat Genet. 2006;38(6):652-658. doi:10.1038/ng1808 16682969

[12] Dess RT, Hartman HE, Mahal BA, . Association of Black race with prostate cancer-specific and other-cause mortality. JAMA Oncol. 2019;5(7):975-983. doi:10.1001/jamaoncol.2019.0826 31120534 PMC6547116

[13] Kodiyan J, Ashamalla M, Guirguis A, Ashamalla H. Race does not affect survival in patients with prostate cancer treated with radiation therapy. Anticancer Res. 2020;40(6):3307-3314. doi:10.21873/anticanres.14313 32487626

[14] Deka R, Parsons JK, Simpson DR, . African-American men with low-risk prostate cancer treated with radical prostatectomy in an equal-access health care system: implications for active surveillance. Prostate Cancer Prostatic Dis. 2020;23(4):581-588. doi:10.1038/s41391-020-0230-6 32327702

[15] Riviere P, Luterstein E, Kumar A, . Survival of African American and non-Hispanic white men with prostate cancer in an equal-access health care system. Cancer. 2020;126(8):1683-1690. doi:10.1002/cncr.32666 31984482

[16] Eschrich SA, Pramana J, Zhang H, . A gene expression model of intrinsic tumor radiosensitivity: prediction of response and prognosis after chemoradiation. Int J Radiat Oncol Biol Phys. 2009;75(2):489-496. doi:10.1016/j.ijrobp.2009.06.014 19735873 PMC3038688

[17] Zhao SG, Chang SL, Spratt DE, . Development and validation of a 24-gene predictor of response to postoperative radiotherapy in prostate cancer: a matched, retrospective analysis. Lancet Oncol. 2016;17(11):1612-1620. doi:10.1016/S1470-2045(16)30491-0 27743920

