# Supplementary material for: Androgen Deprivation Therapy and Outcomes After Radiation Therapy in Black Patients With Prostate Cancer
Source: JAMA Netw Open. 2024 Jun 10;7(6):e2415911. doi: 10.1001/jamanetworkopen.2024.15911 (PMC11165376; doi:10.1001/jamanetworkopen.2024.15911)
Supplement: Supplement 1. — eTable. Cox Regression of Development of Metastases From Time of Biochemical Recurrence eFigure 1. Prostate Cancer Outcomes in all Patients eFigure 2. Kaplan Meier [file jamanetwopen-e2415911-s001.pdf]

Supplemental Online Content

Morgan KM, Riviere P, Nelson TJ, et al. Black race, androgen deprivation therapy, and outcomes after radiation therapy in prostate cancer. *JAMA Netw Open*. 2024;7(6):e2415911. doi:10.1001/jamanetworkopen.2024.15911

eTable 1. Cox Regression of Development of Metastases From Time of Biochemical Recurrence

eFigure 1. Prostate Cancer Outcomes in all Patients

eFigure 2. Kaplan Meier

This supplemental material has been provided by the authors to give readers additional information about their work.

© 2024 Morgan KM et al. *JAMA Network Open.*

eTable 1: Cox Regression of Development of Metastases From Time of Biochemical Recurrence

Variable	Value	HR 95% CI	p value
Black Race		0.86 (0.78-0.94)	<0.001
Ethnicity (reference: Not Hispanic or Latino)	Hispanic or Latino	0.84 (0.68-1.03)	0.094
	Unknown	1.44 (1.03-2.03)	0.034
Charlson (ref: 0)	1	0.69 (0.63-0.75)	<0.001
	≥2	0.78 (0.66-0.92)	0.003
Marital Status	Not married	1.09 (1-1.19)	0.043
Employment Status	Not Employed	0.97 (0.86-1.09)	0.575
Alcohol History	Current	1.01 (0.93-1.11)	0.756
Smoking History	Current	1.01 (0.91-1.12)	0.801
ADT Treatment		1.74 (1.6-1.9)	<0.001
Age at Diagnosis		1.01 (1-1.02)	0.001
Year of Diagnosis (ref: 2000 - 2005)	2006 - 2010	1.30 (1.10-1.55)	0.003
	2011 - 2015	1.31 (1.17-1.45)	<0.001
	2016 - 2020	2.29 (2.03-2.57)	<0.001
MRI		1.50 (1.2-1.87)	<0.001

Abbreviations: HR: hazard ratio, ref: reference group, ADT: androgen deprivation therapy, PSA: prostate specific antigen.

© 2024 Morgan KM et al. *JAMA Network Open.*

eFigure 1: Prostate Cancer Outcomes in all Patients

A: Cumulative Incidence of Biochemical Recurrence

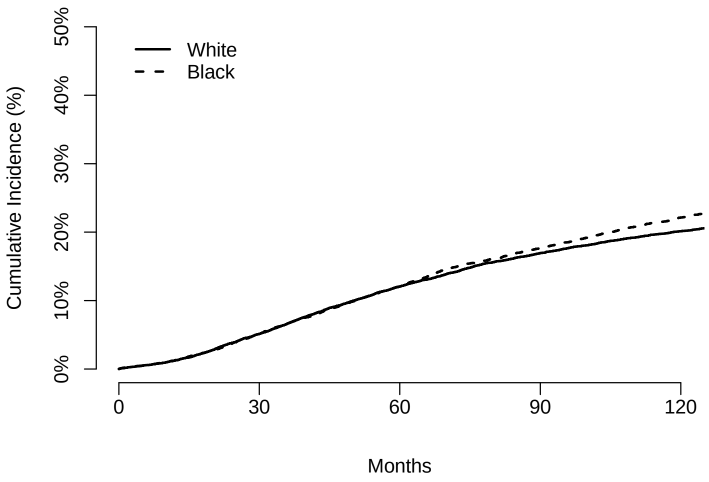

No. at Risk

	0	30	60	90	120
White	17,826	14,998	11,093	7,872	4,993
Black	8,716	7,372	5,416	3,776	2,352

B: Cumulative Incidence of Metastases

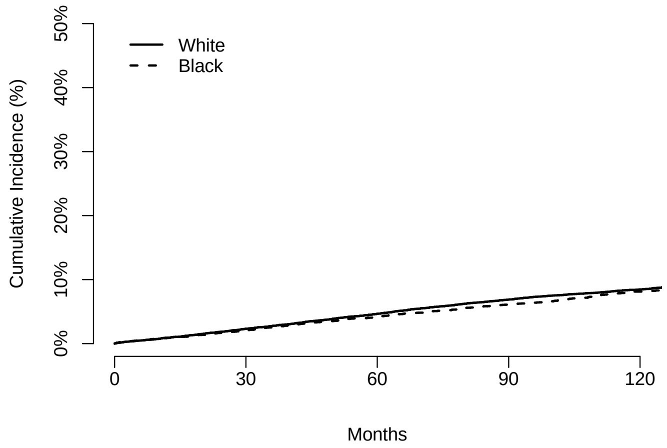

No. at Risk

	0	30	60	90	120
White	17,826	15,444	12,126	8,991	5,947
Black	8,716	7,615	5,955	4,402	2,906

Cumulative incidence function curves of (A) biochemical recurrence and (B) development of metastatic disease from time of completion of radiation therapy, stratified by Black versus White race.

© 2024 Morgan KM et al. *JAMA Network Open.*

eFigure 2: Kaplan Meier

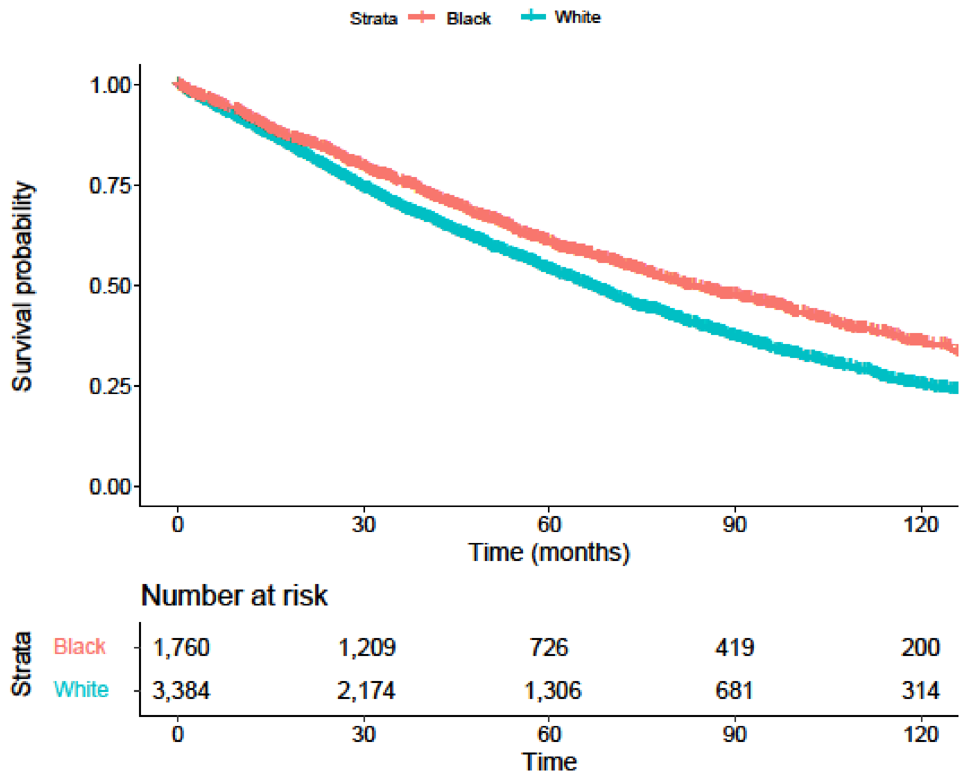

© 2024 Morgan KM et al. *JAMA Network Open.*
