# Supplementary material for: Androgen Deprivation Therapy and Outcomes After Radiation Therapy in Black Patients With Prostate Cancer
Source: JAMA Netw Open. 2024 Jun 10;7(6):e2415911. doi: 10.1001/jamanetworkopen.2024.15911 (PMC11165376; doi:10.1001/jamanetworkopen.2024.15911)
Supplement: Supplement 2. — Data Sharing Statement [file jamanetwopen-e2415911-s002.pdf]

Data Sharing Statement

Morgan. Black Race, Androgen Deprivation Therapy, and Outcomes After Radiation Therapy in Prostate Cancer. *JAMA Netw Open*. Published June 10, 2024.
doi:10.1001/jamanetworkopen.2024.15911

Data
Data available: No

Additional Information
Explanation for why data not available: The data used for this analysis is available to VA Investigators only with proper institutional IRB approval owing to the use of confidential patient information
